# The Synergistic Effects of Polyol Pathway-Induced Oxidative and Osmotic Stress in the Aetiology of Diabetic Cataracts

**DOI:** 10.3390/ijms25169042

**Published:** 2024-08-20

**Authors:** Courtney A. Thorne, Angus C. Grey, Julie C. Lim, Paul J. Donaldson

**Affiliations:** 1 Department of Physiology, School of Medical Sciences, University of Auckland, Auckland 1023, New Zealand; courtney.thorne@auckland.ac.nz (C.A.T.); ac.grey@auckland.ac.nz (A.C.G.); p.donaldson@auckland.ac.nz (P.J.D.); 2 New Zealand National Eye Centre, University of Auckland, Auckland 1023, New Zealand

**Keywords:** diabetic cataract, glucose metabolism, polyol pathway, oxidative stress, osmotic stress, volume regulation

## Abstract

Cataracts are the world’s leading cause of blindness, and diabetes is the second leading risk factor for cataracts after old age. Despite this, no preventative treatment exists for cataracts. The altered metabolism of excess glucose during hyperglycaemia is known to be the underlying cause of diabetic cataractogenesis, resulting in localised disruptions to fibre cell morphology and cell swelling in the outer cortex of the lens. In rat models of diabetic cataracts, this damage has been shown to result from osmotic stress and oxidative stress due to the accumulation of intracellular sorbitol, the depletion of NADPH which is used to regenerate glutathione, and the generation of fructose metabolites via the polyol pathway. However, differences in lens physiology and the metabolism of glucose in the lenses of different species have prevented the translation of successful treatments in animal models into effective treatments in humans. Here, we review the stresses that arise from hyperglycaemic glucose metabolism and link these to the regionally distinct metabolic and physiological adaptations in the lens that are vulnerable to these stressors, highlighting the evidence that chronic oxidative stress together with osmotic stress underlies the aetiology of human diabetic cortical cataracts. With this information, we also highlight fundamental gaps in the knowledge that could help to inform new avenues of research if effective anti-diabetic cataract therapies are to be developed in the future.

## 1. Introduction

Diabetes is characterised by chronic hyperglycaemia that is caused by defects in either insulin secretion, insulin action, or both. Type 1 diabetes is an autoimmune disease that causes the destruction of the insulin-producing pancreatic β cells resulting in insulin deficiency [1]. Type 1 diabetics are usually not obese, and there is a genetic predisposition for the auto-immune destruction of β cells. Type 2 diabetes also has a strong genetic predisposition [2,3], but it is associated with an increasing body mass index [2] and insulin resistance, with a lack of appropriate compensation by β cells leading to insulin deficiency [1]. While the most commonly recognized forms of diabetes include Type 1 and Type 2 diabetes, other forms of diabetes exist including maturity-onset diabetes of the young (MODY). However, cataracts do not appear to be a classic feature and so the overall prevalence of cataract formation in MODY is unknown [4]. On the other hand, a frequent complication of both Type 1 and Type 2 diabetes is cataracts. Cataracts are an opacity of the ocular lens that compromise vision and account for 51% of cases of all blindness, making cataracts the leading cause of impaired vision and blindness globally [5]. The two biggest risk factors for developing cataracts are advancing age and diabetes [6], with diabetic patients being up to five times more likely to develop cataracts, and at an earlier age than non-diabetics [7,8].

Currently, the only treatment for either age-related or diabetic cataract involves the surgical removal of the cataractous lens, usually by phacoemulsification, and replacement with an intraocular plastic implant [9,10]. However, with an aging population and an increasing incidence of diabetes, the demand for cataract surgery has increased, and it is now the most commonly performed surgical procedure in the world [11]. As a result, public health systems are under extreme pressure with wait times for cataract surgery commonly exceeding 3 months in European nations [12,13], rising to upwards of 6 months [14] or even several years in other countries [15,16]. An increased wait time for cataract surgery is associated with greater vision loss, an increased rate of falls, and an overall lower quality of life [14]. For diabetic patients with cataracts, the risk of complications associated with cataract surgery are higher than the general population. These risks include enhanced progression of diabetic retinopathy [17], the development of photic retinopathy [18], and macular oedema [19]. More complex surgical procedures are also required to manage the additional risks for diabetic patients with cataracts [20] and post-surgical healing is reported to be slower in these patients [21,22]. Diabetic patients with cataracts also experience more issues with replacement lenses, and the options are more limited due to possible contraindications with the materials that some intraocular implants are made from [23,24,25,26]. For these reasons, there has been an intense effort to develop medical therapies to delay the onset and/or progression of diabetic cataracts and reduce the burden on global healthcare systems.

The incidence of cortical and/or posterior-subcapsular cataracts (PSC) is higher in diabetic patients compared with non-diabetics, with an earlier onset, and progression of cataracts being more rapid [20]. Although other forms of cataract can and do present in diabetic patients, cortical cataracts tend to be the more prevalent form [7]. While detailed morphological analysis on human diabetic cataract lenses are lacking, analysis of animal models indicates cell morphology is disrupted in the cortex of diabetic lenses. Questions remain on how the elevated glucose levels associated with diabetes evoke these localised changes in lens fibre cell volume that manifest as cell swelling and opacification in the cortex. The work on diabetic rat models initially suggested that the observed loss of cell volume regulation was the result of osmotic stress due to the accumulation of sorbitol, an impermeable osmolyte formed from the metabolism of excess glucose via the polyol pathway. As the accumulation of sorbitol attracts fluid, this results in the cell swelling and tissue liquefaction observed in these animals [27]. Based on these animal studies, considerable attention was focussed on the development and testing of inhibitors of aldose reductase (AR), the enzyme that converts excess glucose to sorbitol [28]. However, while these inhibitors have proven to be very successful in ameliorating diabetic cataracts in rats and dogs [29,30,31,32,33,34], they proved ineffective as anti-cataract therapies in human diabetic cataract patients [33,35]

The differential effectiveness of AR inhibitors on cataract progression between rats and humans has been attributed to species differences in AR activity, and other differences in the metabolism of excess glucose. What is not yet sufficiently addressed is the question of how these differences may impact the underlying physiology that normally maintains transparency to lead to the diabetic cataract phenotype seen in human patients. This is not a systematic review proper, but instead a review in which we have focused on identifying pathways, old and new, that contribute to cataract formation and examining how they differ between animal and human lenses to provide a greater understanding of the pathobiology of diabetic cataracts. This has led to identifying gaps in our knowledge and hence areas of future research which may promote further research on the topic and assist the goal of developing improved therapies to prevent the looming diabetic cataract epidemic.

To provide a context for this current review, we first describe the diabetic cataract damage phenotype before providing an overview of how the structure and function of the lens results in variations in the uptake and metabolism of glucose in the morphologically distinct regions of the lens. We provide an overview of the physiological adaptations that maintain lens transparency that are vulnerable to damage during hyperglycaemia, before discussing how the biochemical changes arising from altered glucose metabolism in hyperglycaemia may impact these adaptations, eventually manifesting as the distinctive cortical cataract phenotype observed in human diabetic cataract patients.

## 2. The Diabetic Cataract Phenotype

**Figure 1:**
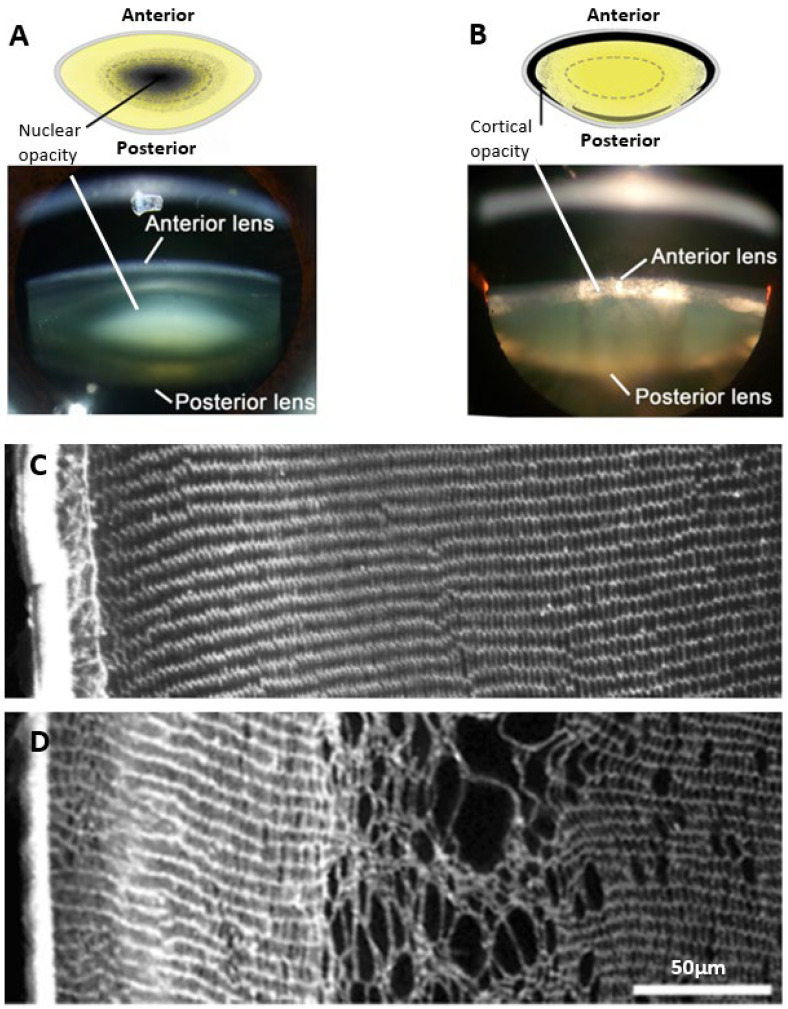
Regional and morphological characteristics of age-related nuclear and diabetic cortical cataracts. (**A,B**) Schematics (**top panel**) and Scheimpflug slit-lamp photographic images (**bottom panel**) showing the two main types of cataracts: (**A**) nuclear cataract and (**B**) cortical cataract. (**C**) Rat lens section stained with wheat germ agglutinin (WGA) to highlight lens morphology, showing the regular and organised architecture of the outer cortex. (**D**) Streptozotocin rat lens section stained with WGA highlighting the disrupted fibre cell morphology and cell swelling in a distinct region within the outer cortex of the lens. Figure (**A**,**B**), (**top panels**); Adapted from Lim, J. C. et al. (2020) [42] under the open access Creative Commons CC BY 4.0 license. Figure (**A**,**B**), (**bottom panels**); Adapted with permission from Lim, J. C. et al. (2017) [43]. Figure (**C**,**D**); Reproduced with permission from Donaldson et al. (2009) [44].

The damage phenotypes associated with age-related and diabetic cataracts are distinctly different. Age-related cataracts typically result in opacification of the lens nucleus due to modification of proteins and protein aggregation, without any obvious signs of changes to lens morphology (Figure 1A). On the other hand, in diabetic cataracts the opacification initially appears in the outer lens cortex (Figure 1B) with changes in fibre cell morphology evident [36]. Due to the phacoemulsification technique used in cataract surgery where the lens is fragmented, very few studies have been able to examine at the cellular-level localised morphological changes occurring in intact human diabetic cataractous lenses. One early study examined two human diabetic cataractous lenses using transmission electron microscopy and reported extensive morphological damage, especially at the cortical–nuclear interface [37]. The presence of multilamellar membrane aggregates, loss of cytoplasmic material, and irregularity in the shape and packing of fibre cells was also noted in the deeper cortex with no changes in fibre cell morphology in the nuclear regions; however, no analysis of the cortical region was performed. Due to the lack of detailed histological data in humans, information from animal studies has typically been used to inform our understanding on the mechanisms that may initiate the damage observed in diabetic cortical cataracts. The most widely used model is the streptozotocin (STZ) rat, in which Type 1 diabetes is induced by chemically destroying the insulin-secreting pancreatic β cells [38]. These animals exhibited signs of morphological damage in the outer cortex, the same region in which opacities in human diabetic cataracts most commonly occur (Figure 1C,D). Immunohistochemistry experiments revealed cell swelling and tissue liquefaction in a distinct layer of fibre cells 150–200 µm from the lens surface [39]. In another rat model of diabetes in which animals are fed a high galactose diet intended to mimic Type 2 diabetes [38], there was the appearance of anterior subcapsular vacuoles and associated swelling, disorganisation, and liquefaction of fibres initially in the equatorial regions, which then progressed towards the anterior and posterior cortical regions of the lens [40,41]. Taken together, these studies all demonstrate cell morphology to be disrupted in the cortex of diabetic lenses. However, what these morphological studies do not show is in what way hyperglycaemia is altering the underlying physiology that normally maintains the transparent and refractive properties in this region of the lens.

## 3. The Lens Is Adapted for Transparency

**Figure 2:**
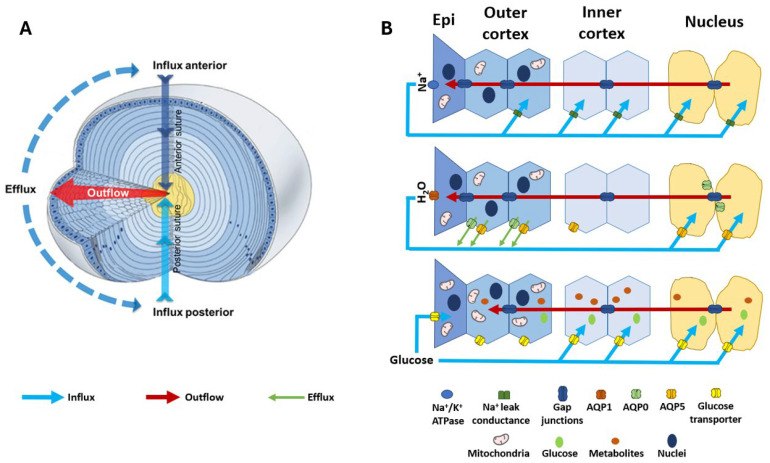
Structure and function of the lens. (**A**) 3D representation of the lens microcirculation system. Ion and fluid fluxes enter the lens at both poles and travel via the extracellular spaces (blue arrows) before crossing cell membranes and travelling via a gap junction-mediated intracellular route to exit the lens at the equator (red arrows). (**B**) Schematic detailing the regional distribution of ion channels, water channels, and glucose transporters that facilitate the movement of Na^+^ (**top panel**), fluid (**middle panel**), and glucose (**bottom panel**) in the microcirculation system. (**Top panel**): Na^+^ is actively removed from the lens by the Na^+^/K^+^ ATPase and re-enters at the poles. Na^+^ is taken up from the extracellular space in the deeper regions of the lens via Na^+^ leak channels before returning to the surface via a gap junction-mediated intracellular route. (**Middle panel**): Water follows the movement of Na^+^, travelling from the core to the surface via gap junctions and exiting the lens surface via aquaporin (AQP) water channels. Regional distributions in AQP channels with varying permeability together with a hydrostatic pressure gradient allow water to exit fibre cells in the periphery and re-enter fibre cells in the nucleus. (**Bottom panel**): Glucose uptake is mediated by glucose transporters expressed throughout the lens, enabling fibre cells to directly take up extracellular glucose in all regions of the lens. While in epithelial cells at the lens surface this uptake occurs directly from the surrounding humours, in deeper fibre cells the glucose is delivered to mature fibre cells by the microcirculation system. Once inside the cell, glucose can be utilised in many metabolic processes to release energy in the form of ATP, which is required to maintain the structural integrity and transparency of the lens. Adapted with permission from Donaldson PJ et al. (2003) [50].

To understand how localised disruptions in lens morphology occur under hyperglycaemic conditions and how these changes manifest as opacity and cataract, we first need to appreciate how the transparent and refractive properties of the lens are established and maintained under normal conditions. This is achieved by a specialised tissue architecture that is actively maintained by a unique cellular physiology [45] (Figure 2A). The anterior surface of the lens consists of a single layer of epithelial cells which at the lens equator divide and begin a process of cell differentiation and internalisation to form highly elongated secondary lens fibre cells, which comprise the outer cortex of the lens. During this process of differentiation, cell nuclei and other organelles such as mitochondria are degraded in order to remove these light-scattering elements from the light pathway [46,47]. In addition, these differentiating fibre (DF) cells express new proteins including crystallins which contribute to the formation of the gradient of refractive index (GRIN) that forms the basis of the refractive properties of the lens [48]. As DF cells lose their organelles and become further internalised, they become mature fibre (MF) cells and form the inner cortex of the lens. The inner cortex in turn surrounds the primary fibre (PF) cells that were initially laid down in utero and which form the nucleus, or core, of the lens [49]. The loss of intracellular organelles means that only the most peripheral cells of the outer cortex are capable of protein synthesis and aerobic metabolism, while mature fibre cells are not able to synthesise new proteins and rely on anaerobic metabolism to meet their energy requirements. The fibre cells are also lifelong, making the tissue susceptible to the chronic accumulation of damage to structural and functional proteins. The continual process of epithelial cell division, fibre cell differentiation, and the internalisation of maturing cells means that the lens grows throughout life, and that a gradient of fibre cells of increasing age, and with distinctly different properties and metabolic demands, exists in the lens.

### 3.1. Water Transport Is Fundamental to Transparency

In the lens outer cortex, light scatter is prevented by the ordered arrangement of the fibre cells which pack tightly into a repeating geometric arrangement [51]. The cross sectional dimensions of cortical fibre cells remain constant between many species [52], underlining the importance of the regularity of the cellular lattice. This repetitive spatial order creates a regular variation in refractive index between the cytoplasmic and cell membrane components of the fibre cells, preventing light scatter due to an optical phenomenon known as the diffraction grating effect [53]. In contrast, the lens nucleus contains mature fibre cells that have lost their regular ordered morphology. In this region of the lens, light scatter is reduced due to the expression of higher levels of crystallin proteins than cortical fibre cells, which matches the refractive index between membranes and the cytoplasm [53,54]. What is common between these two morphologically distinct regions is the need to maintain stringent control of the water content if lens transparency is to be maintained. In the unordered lens nucleus, the buffering of the water content serves to eliminate any local transient mismatches between the refractive index of the membranes and cytoplasm, while in the outer cortex any failure to maintain the water content will change the fibre cell volume, resulting in disruption to the precise spatial organisation of the fibre cells, resulting in light scatter that compromises transparency—a damage phenotype that is seen in diabetic cortical cataracts [44]. Hence, while it is the structural organisation of the lens that establishes its transparent and refractive properties, the active regulation of water transport is required to maintain these properties [45].

### 3.2. The Lens Microcirculation System

In the absence of a blood supply, the lens operates an internal microcirculation system which generates a circulating flux of ions and water (Figure 2A) that serves not only to control the lens water content and fibre cell volume, but also to deliver nutrients such as glucose to the lens nucleus faster than would occur by passive diffusion. Regional differences are evident in the distribution of the transporters and water channels that facilitate the fluid and ion fluxes of the microcirculation (Figure 2B, middle and top panel) [55,56,57,58], which may reflect the regional specificity of diabetic cortical cataracts. The importance of the function of these transporters in maintaining lens transparency is seen where the mutation of aquaporin 0 (AQP0) in humans causes congenital cataracts [59,60]. Since the molecular and cellular components that generate and regulate this system have been extensively reviewed [45,50,61,62] and are summarised here in Figure 2, we will mainly focus on the components that are relevant to the aetiology of diabetic cataracts, the control of fibre cell volume in the outer cortex, and regional differences in the delivery and subsequent metabolism of glucose.

## 4. Regulation of Lens Water Content and Cortical Fibre Cell Volume

The circulating Na^+^ current generated by the spatial difference in Na^+^ pumps and leak channels (Figure 2B, top panel) drives a net flux of solutes that generates local osmotic gradients and drives the transport of water through the lens (Figure 2B, middle panel). These fluxes of ions and water preferentially enter the lens at both poles via an extracellular pathway, then cross into fibre cells deeper in the lens before leaving the central lens via an intracellular outflow pathway mediated by gap junction channels. The subcellular organisation of these gap junctions serve to direct ion and fluid flow towards the equatorial efflux zone of the lens where the channels and transporters that mediate their exit from the lens are concentrated (Figure 2B, middle panel). This flow of water through the lens in turn delivers nutrients such as glucose into the lens nucleus faster than would occur by passive diffusion alone. Glucose is then taken up from the extracellular space by glucose transporters expressed in the membranes of fibre cells before being metabolised by the different metabolic pathways active in the different lens regions (Figure 2B, bottom panel). While this flow of ions and water throughout the lens has been shown to be necessary for the maintenance of the transparency and refractive properties of the lens, at the local cellular level this flow of water through fibre cells has the potential to alter the volume of individual fibre cells. If not compensated for, this would alter the local spatial order that establishes lens transparency in the outer cortex. Hence, the lens requires a regulatory system that integrates the modulation of water flow throughout the lens at the whole tissue level, with local control of fibre cell volume to ensure that fibre cell morphology in the outer cortex is maintained in order to support transparency.

### 4.1. Whole Lens Volume Regulation

It is well established that the lens can dynamically regulate its water content in response to an imposed osmotic challenge [45,63,64,65] and that differentiating fibre cells in the outer cortex utilise a variety of ion channels and transporters to actively maintain their individual cell volume (and therefore the overall volume of the lens) in response to an osmotic challenge [44,66]. Lenses exposed to hypo-osmotic challenge exhibit an initial swelling before undergoing a regulatory volume decrease (RVD) mediated by the efflux of K^+^ and Cl^−^ ions and an associated loss of internal water, reducing lens volume. In contrast, lenses placed in a hyperosmotic media undergo an initial shrinkage, which is subsequently restored by a regulatory volume increase (RVI) driven by the intracellular accumulation of K^+^, Na^+^, and Cl^−^ ions and consequent water influx [63,64,65]. Subsequent studies on rat lenses cultured under isosmotic conditions in the presence of reagents that modulate the activities of the cation chloride cotransporters (CCCs) and other chloride channels [67] have shown that a constitutively active flux of Cl^−^ ions exists in the lens cortex that regulates the steady state fibre cell volume [44]. The CCCs include the sodium–potassium–chloride cotransporter (NKCC1) and potassium chloride cotransporters (KCC1-4).

### 4.2. Fibre Cell Volume Regulation

**Figure 3:**
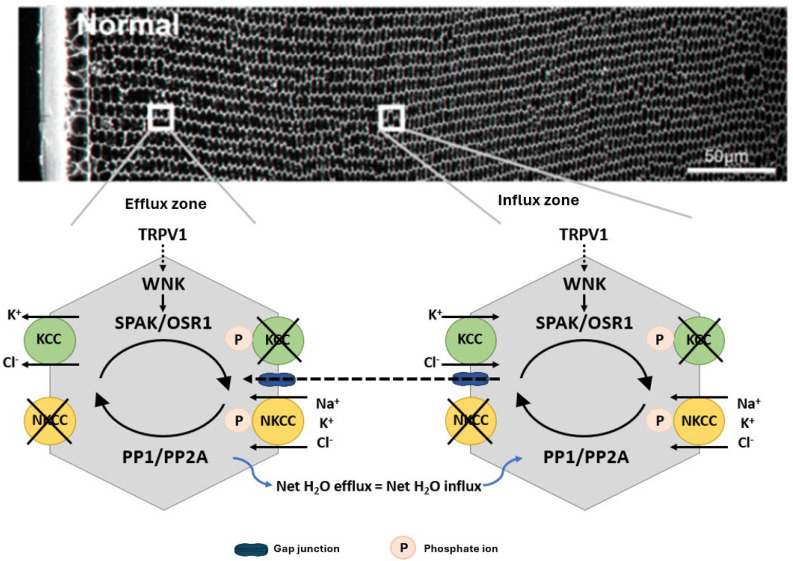
Regulation of fibre cell volume in the lens. In the normal lens, the direction of ion gradients promotes ion influx mediated by KCC and NKCC in deeper cortical fibre cells. Gap junctions connect these deeper cells to a peripheral zone where KCC efflux predominates. By modulating their phosphorylation status, the activity of the transporters is reciprocally regulated so that influx equals efflux and lens volume is maintained. TRPV1 has been shown to respond to hyperosmotic-induced cell shrinkage by inducing the phosphorylation and activation of NKCC1 to promote a regulatory volume increase response that restores lens volume. Adapted with permission from Donaldson PJ et al. (2017) [45].

In other cell types, the activity of NKCC1 and KCCs are reciprocally regulated by a signalling pathway that modulates their phosphorylation status [68] to ensure that at steady state the cell volume is held constant (Figure 3). This pathway involves members of the With No Lysine Kinases (WNK1 to 4) family [69] that respond to osmotic stress by phosphorylating and activating two closely related kinases; the Ste-20 like Proline Alanine Rich Kinase (SPAK) and Oxidative Stress Response Kinase 1 (OXSR1) [70]. These kinases directly phosphorylate both NKCC1, causing its activation, and KCC, causing its inactivation. The phosphorylation of these cotransporters is reversible through the actions of the phosphatases PP1 and PP2A, which therefore deactivate NKCC1 and activate KCC. The molecular and functional identification of CCC transporters [71,72,73,74] together with their upstream WNK-SPAK/OSR1 kinase signalling pathways [75,76] shows that the lens operates a similar regulatory system to control fibre cell volume (Figure 3). More recently, hyperosmotic-induced cell shrinkage has been shown to activate a signalling pathway initiated by the activation of the mechanosensitive transient receptor potential vanilloid-1 (TRPV1) channel. TRPV1 activation leads to the phosphorylation-dependent activation of NKCC1 to induce an RVI response that restores lens volume [75,77]. Hypo-osmotic stress activates a different mechanosensitive non-selective cation channel, TRPV4, which triggers the efflux of ATP to the extracellular space [78,79], thereby activating P2Y receptors that in turn increase Na^+^/K^+^ ATPase activity through an Src family kinase-dependent pathway in the lens. This also forms part of the RVD response to alleviate the cell swelling induced by the hypo-osmotic environment. Taken together, it is apparent that the lens has all the machinery necessary to regulate fibre cell volume in response to osmotic challenges.

Interestingly, it now appears that many of the transducers, signalling pathways, and effectors involved in regulating cell volume in response to a change in osmolarity are also involved in modulating the flow of water through the whole lens. This intracellular flow of water through the gap junction-mediated outflow pathway generates a substantial hydrostatic pressure gradient [80]. Changes in the lens surface pressure are sensed by the mechanosensitive cation channels TRPV1 and TRPV4, whose activity mediates signalling pathways that reciprocally regulate lens water transport to maintain the pressure gradient as well as regulating individual fibre cell volume [77,81,82]. Thus, it appears that both water flow through the lens and the localised control of fibre cell volume are integrally regulated. It follows, therefore, that any dysregulation of this system caused by hyperglycaemia would manifest as the localised fibre cell swelling observed in diabetic cortical cataracts. What is currently not well established, particularly in the human lens, is how the elevated glucose levels in diabetes impact the ability of the lens and the cortical fibre cells to regulate their volume.

## 5. Regional Differences in Glucose Delivery and Metabolism

Given the regional specificity of diabetic cataracts to the lens outer cortex as well as a clear link to hyperglycaemic glucose metabolism, it is suggested that regional differences exist in the impact of and/or response to elevated glucose. Understanding these differences may be the key to fully understanding the aetiology of diabetic cataracts. Insights into how glucose is delivered to, taken up by, and metabolised in the different regions of the normal lens has only recently begun to be studied [83,84]. In this section, we review these processes to provide a basis for discussion on how they may be altered under the conditions of elevated glucose observed in diabetes.

### 5.1. Glucose Uptake and Delivery

The primary source of energy for the lens is glucose. As the lens is avascular, glucose must be taken up from the surrounding ocular humours. In humans, the concentration of glucose is 3.2 mM in the aqueous humour [85], and 3.0 mM in the vitreous humour [86]. The normal concentration of glucose in the lens is in the region of 10 mg/100 g of tissue, though some species variation does exist [87]. It was previously thought that the uptake of glucose from the surrounding humours only occurred via the anterior epithelial cells and that the delivery of glucose throughout the lens occurred by passive diffusion by an intracellular route mediated by gap junctions [88]. We now know that the lens operates a microcirculation system which drives the delivery of nutrients such as glucose to the lens nucleus faster than by passive diffusion (Figure 2) [89,90]. In support of this, imaging mass spectrometry used to visualise the time course of movement of isotopically labelled ([U–^13^C]) glucose into different regions of organ cultured bovine lenses [83] revealed an initial glucose uptake occurring in the equatorial region of the lens followed by a secondary delivery of the labelled isotope to the deeper layers of the lens. This occurred at a rate faster than would be predicted via passive diffusion alone [83], and it could be partially inhibited by inhibition of the microcirculation system (unpublished data).

Consistent with the microcirculation-based model of the extracellular delivery of glucose to deeper fibre cells (Figure 2B, bottom panel) is the expression of glucose transporters throughout the lens [91,92], suggesting that the uptake of glucose from the extracellular space can occur in all lens regions. Glucose uptake occurs via two major protein families; the facilitative GLUT transporter family which mediates the passive movement of glucose down its diffusion gradient [93], and the secondary-active SGLT transporter family which utilises the transmembrane electrochemical gradient of Na^+^ to drive the uptake of glucose. GLUT and SGLT isoforms have been examined in the lenses of several species including rats, mice [92,94], humans, and bovines [91] and revealed species differences in the expression of GLUTs and SGLTs. In rat lenses, GLUT1 was expressed in the epithelium and outer cortex, while GLUT3, SGLT1, and SGLT2 were identified in the deeper regions [91]. Bovine lenses mainly expressed GLUT3 in the epithelium and GLUT1 in fibre cells. The more abundant expression of GLUT1 in the equatorial region of the bovine lens was consistent with the view that glucose uptake in the lens cortex was primarily driven by GLUT1-mediated uptake [83,91]. In human lenses, GLUT1 but not GLUT3 was initially detected [91]. More recently, a third GLUT isoform, GLUT12, has been discovered in the human lens where it was shown to be expressed throughout the lens, with its abundance decreasing with increasing age [95]. In skeletal muscle, GLUT12 is insulin-sensitive [96], suggesting that in the lens GLUT12 may be responsible for insulin-sensitive glucose uptake.

### 5.2. Glucose Metabolism Pathways

The unique structure and function of the lens creates metabolic compartments with varied metabolic demands and capabilities, and this reflects the regional specificity of diabetic cortical cataracts. In the lens, glucose is metabolised by three pathways; glycolysis [97], the pentose phosphate/hexose monophosphate shunt pathway [98], and the polyol pathway [99]. Only peripheral fibre cells that still contain mitochondria are capable of aerobic metabolism, and it was previously thought that this was the primary route of lenticular glucose metabolism and ATP production. However, further studies have demonstrated that only around 3% of glucose is consumed via aerobic metabolism [100,101], accounting for only around 20–30% of ATP production. This means that the remaining majority of ATP production arises from anaerobic glucose metabolism [37] and that this may occur throughout all lens regions. This is important as, in the lens, glucose metabolism via the pentose phosphate pathway serves another essential function aside from ATP production; the metabolism of glucose-6-phosphate (G6P) by the enzyme glucose-6-phosphate dehydrogenase generates the cofactor NADPH, which is essential for maintaining redox balance in the lens by regenerating reduced glutathione (GSH) from its oxidised form, GSSG [98]. Compartmentalisation of different metabolic pathway enzymes is also evident. Hexokinase is predominantly active in the epithelium of the adult human lens [102] and is almost entirely absent from mature fibre cells [103]. In the polyol pathway, glucose is metabolised first to sorbitol by the enzyme aldose reductase (AR), and then from sorbitol to fructose by the enzyme sorbitol dehydrogenase (SDH). In human lenses, AR is found predominantly in the epithelium [104], whereas SDH is also found in the lens cortex even in adult lenses. The relative activity of these enzymes also differs between species, where AR activity is relatively much lower in humans compared to animal lenses, whereas SDH activity is relatively higher [104]. Mapping the metabolism of [U–^13^C] glucose in bovine lenses, Zahraei et al. [83] revealed that in the cortex, [U–^13^C] glucose was rapidly metabolised, forming several compounds that could be spatially localised by imaging mass spectrometry. In addition, [U–^13^C] glucose and some [U–^13^C] metabolites such as [U–^13^C] sorbitol were detected in the lens nucleus after 20 h of incubation which is considerably shorter than is predicted by passive diffusion [105], indicating regional differences in the pathways used to metabolise glucose in the lens [83]. Hence, it appears that regional differences in delivery, uptake, and metabolism occur in the normal lens exposed to physiological levels of glucose. In the next section, we investigate what is known about how hyperglycaemia affects glucose metabolism in the whole lens by focussing on the polyol pathway and how elevated glucose levels differentially affect the different regions of the lens and contribute to osmotic and oxidative stress.

## 6. Effect of Hyperglycaemia on Glucose Metabolism: A Focus on the Polyol Pathway

The pathological effects of chronic hyperglycaemia on the lens have been variously attributed to an increased flux of glucose through the polyol pathway and/or the direct effects of glucose on the formation of advanced glycation end products (AGEs). While data from animal studies have focused on osmotic stress arising from increased polyol metabolism of glucose, both AGE formation and polyol metabolism can compromise antioxidant defence systems, generate ROS, and compromise lens structure and function [106,107,108]. Here, we will discuss how the increased flux of glucose through the polyol pathway in hyperglycaemia can impact the redox environment of the lens, how this can impair lens function and transparency, and the evidence for the contribution of these effects to diabetic cataractogenesis.

### 6.1. The Polyol Pathway

**Figure 4:**
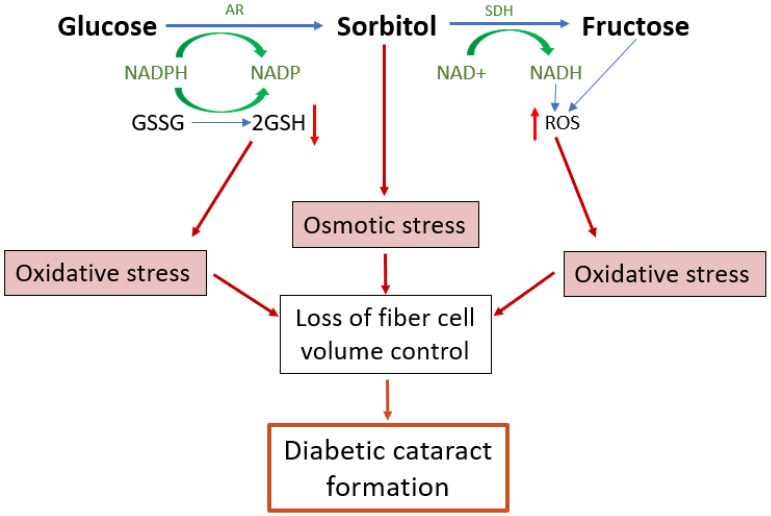
Emerging model of the contribution of polyol pathway-induced osmotic stress and oxidative stress to diabetic cataract formation. The polyol pathway is a non-rate limited pathway in which glucose is converted first to sorbitol and then from sorbitol to fructose. Upregulation of the polyol pathway activity in diabetic lenses leads to both osmotic and oxidative stresses and the formation of reactive glycating metabolites. The accumulation of sorbitol attracts fluid, leading to cell swelling. Reduced antioxidant capacity and the increased formation of ROS both increase oxidative stress which in turn can result in dysfunction of the cell volume machinery of the lens, causing cell swelling and light scatter. AR *=* aldose reductase, SDH *=* sorbitol dehydrogenase, GSH *=* reduced glutathione, GSSG *=* oxidised glutathione, ROS *=* reactive oxygen species.

Under hyperglycaemic conditions, the hexokinase pathway becomes saturated and excess glucose is instead channelled into the polyol pathway [109]. In the polyol pathway, glucose is converted to sorbitol by the enzyme aldose reductase (AR)—a reaction that depends on the donation of an electron from the cofactor NADPH which then becomes NADP^+^ (Figure 4). Sorbitol is then further metabolised to fructose by the enzyme sorbitol dehydrogenase (SDH), utilising 2 molecules of NAD^+^ which accept 2 electrons to become 2NADH. As much as 30% of glucose enters the polyol pathway in hyperglycaemic conditions [109]. This upregulated polyol activity induces a number of direct and downstream stressors that impact on the ability of lens to regulate the volume of its fibre cells (Figure 4), which ultimately manifests as a diabetic cataract in the lens cortex.

### 6.2. Direct Effects of Sorbitol as an Osmotic Stressor

Sorbitol is an impermeable osmolyte that cannot cross cell membranes, so its accumulation imposes a direct osmotic stress that promotes cell swelling [27]. The role of sorbitol as an osmotic stressor contributing to cell swelling in diabetic cataracts is well established from animal models such as the streptozotocin (STZ) rat model. In this model of Type 1 diabetes, the phenotype of cell swelling and tissue liquefaction in the lens outer cortex [39] is prevented by treatment with aldose reductase inhibitors that prevent the accumulation of sorbitol [34]. While also showing success in canines [33] this treatment has proven unsuccessful in humans [33,35], highlighting species differences in diabetic cataract aetiology. This may be explained by the fact that AR activity in rats is higher relative to SDH, while in humans SDH activity is higher relative to AR [104]. An increased flux of glucose through the polyol pathway would therefore favour fructose production in humans, indicating that the physiological impact of fructose and its metabolites may play a larger role in the pathogenesis of human diabetic cataracts. Furthermore, while the STZ rat model has achieved much attention, a great degree of species differences exist across different animal models, as reviewed by Lim et al. [43]. Although a common trait in these models, as well as humans, is opacification and morphological changes in the outer cortex, the exact phenotype, time to onset, and degree of damage varies greatly. Lastly, it is important to note that the appearance of diabetic cataracts in humans occurs over several decades, whereas animal models can develop sorbitol-induced cell swelling and opacification within weeks or even days [110,111]. These inconsistencies highlight two important things. Firstly, the formation of diabetic cataracts cannot be ascribed universally to osmotic stress from sorbitol accumulation alone. Secondly, in the lenses of different species there must exist differences in either the metabolism of glucose in hyperglycaemia and/or the response to the stresses this induces.

### 6.3. The Polyol Pathway and Fructose Metabolites Generate Oxidative Stress

The polyol pathway introduces oxidative stress to the tissue through several mechanisms, including the consumption of NADPH, the accumulation of NADH, and the metabolic and physiological changes that result from downstream fructose metabolism, such as protein glycation and irreversible AGE formation [112]. As the cortical cell swelling in human diabetic cataracts cannot be solely attributed to the osmotic stress from sorbitol accumulation, the impact of the oxidative stress and protein glycation that result from increased polyol metabolism and fructose production likely plays a role in diabetic cataractogenesis that is currently understudied. We propose that a synergistic effect of osmotic stress, oxidative stress, and protein glycation results in the formation of diabetic cataracts in humans by chronically impairing the volume regulatory responses of the lens fibre cells (Figure 4), and we discuss below the evidence for this mechanism occurring in the diabetic lens.

### 6.4. Altered Redox Balance Induced by Increased Flux of Glucose through the Polyol Pathway

The conversion of glucose to sorbitol by AR consumes NADPH (Figure 4), a cofactor required to regenerate the principal lens antioxidant glutathione (GSH) from its oxidised form (GSSG) [82] (Figure 4). Increased polyol pathway activity during hyperglycaemia will therefore deplete the amount of GSH and expose the lens to oxidative stress. Consistent with this is that GSH levels are reduced in diabetic vs. non-diabetic cataractous lenses [113], and the level of NADPH is reduced by around 15% in diabetic lens models [114]. The pentose phosphate pathway is the main regenerative source of NADPH and would normally be expected to restore NADPH levels. However, the production of NADPH is the rate limiting step of the pentose-phosphate pathway, while the consumption of NADPH by aldose reductase is not rate limited. Hence, an increase in glucose flux through the polyol pathway would be expected to overwhelm the capacity of the pentose phosphate pathway to maintain the supply of NADPH needed for GSH regeneration. This view is further supported by the observation that the activity of glucose-6-phosphate dehydrogenase, the key regulatory enzyme of the pentose phosphate pathway, is decreased in the diabetic lens [115,116]. Other signs of increased oxidative stress in the diabetic lens includes increased lipid peroxidation products [117], an increase in oxidised glutathione (GSSG), and the depletion of other non-enzymatic antioxidants such as ascorbate and taurine [118], as well as a decrease in the activity of antioxidant defence enzymes such as superoxide dismutase, catalase, and glutathione peroxidase [115,119].

**Figure 5:**
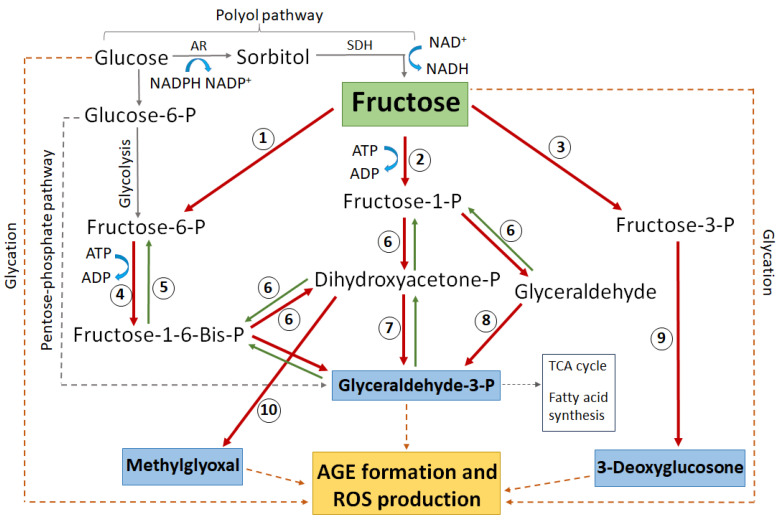
Schematic highlighting the major glucose and fructose metabolic pathways that lead to protein glycation, AGE formation, and subsequent ROS generation. Enzymes numbered in circles: 1 *=* hexokinase, 2 *=* ketohexokinase (aka fructokinase), 3 *=* fructose-3-phosphokinase, 4 *=* phosphofructokinase, 5 *=* fructose-1-6-bisphophatase, 6 *=* aldolase, 7 *=* triose-phosphate isomerase, 8 *=* triose kinase, 9 *=* hydrolysis by unknown enzyme, 10 *=* methylglyoxal synthase. AR *=* aldose reductase, SDH *=* sorbitol dehydrogenase, P *=* phosphate. Blue boxes indicate the most reactive glycating metabolites. The dashed grey line indicates complex pathways without all intermediate metabolites shown. The dashed brown line indicates direct non-enzymatic glycation.

The subsequent conversion of sorbitol to fructose contributes to oxidative stress through the generation of NADH (Figure 4). Raised levels of NADH affect the redox environment in a number of ways, and to such a degree that the change in the ratio of NADH to NAD^+^ is referred to as ‘pseudohypoxia’ [120]. For instance, NADH can act as a substrate for the enzyme NAD(P)H oxidase (NOX), which catalyses electron transfer from NADH to oxygen resulting in the production of superoxide, a highly reactive ROS. Excess NADH will also push more electrons into the mitochondrial electron transport chain, where these are donated by co-enzyme Q to molecular oxygen, again generating superoxide [121]. Moreover, an increase in NADH will inhibit glycolysis (83), limiting the availability of pyruvate for the regeneration of NAD^+^ and thereby maintaining the imbalance in the ratio of NAD+:NADH. Due to the inhibition of glycolysis, more glucose will also enter the polyol pathway, which again will maintain the altered NAD^+^:NADH ratio. As such, these effects will compound each other to further entrench the tissue in a state of oxidative stress. Furthermore, the downstream metabolism of fructose will further contribute to ROS production by consuming ATP, both in the conversion of fructose to fructose-1-phosphate (F-1-P), and in the conversion of fructose-6-phosphate to fructose-1,6-bisphophate (Figure 5; Steps 2 and 4). Lowered ATP levels stimulate mitochondrial respiration [122], which will again increase superoxide production. Importantly, the consumption of ATP in conversion of fructose to F-1-P occurs rapidly as it is not rate limited [123]. Also, and of specific importance in the lens, GSH synthesis is a highly ATP-dependent process, and so the reduction in ATP levels may impair GSH synthesis, further compromising the redox balance. It is important to note the involvement of mitochondria in several of these processes, which is undoubtedly linked to the regional specificity of the diabetic cataract phenotype.

### 6.5. Formation of Advanced Glycation End Products (AGEs) by Fructose and Fructose Metabolites

In a variety of tissues, it has been shown that diabetic-associated hyperglycaemia results in the formation of advanced glycation end products (AGEs) [84]. AGEs are generally formed via non-enzymatic condensation between the carbonyl groups of a reducing sugar (or sugar alcohol) and a free amine group of a protein [124]. AGEs induce oxidative stress through a number of mechanisms. The binding of AGEs to their receptor, RAGE (receptor for AGEs), activates the enzyme NOX via Protein Kinase C [125] leading to the production of superoxide, hydrogen peroxide, and hydroxyl radicals [126,127,128]—a process strongly linked with oxidative damage in diabetes [129]. AGE/RAGE binding also activates the transcription factor NF-κB, which in turn induces further RAGE expression, creating a positive feedback loop that further increases the responses that generate ROS [130,131]. In the lens epithelial cells of human diabetic patients, RAGE expression is upregulated [132] indicating higher levels of AGEs. We also know that NOX, the enzyme that is activated by AGE/RAGE binding to generate several ROS, is present in the human lens. In human and rabbit lens epithelial cells, five isoforms of the NOX family (NOX1, NOX2, NOX3, NOX4, and NOX5) have been identified [133]. Currently, it is unknown whether one specific isoform contributes more to ROS generation under hyperglycaemic conditions.

While exposure to high glucose can directly increase AGE formation (Figure 5, dashed brown lines), the rate at which a sugar can glycate a protein is directly proportional to the amount of the sugar existing in an open chain format. As a result, fructose and its metabolites are much more potent glycating agents than glucose [134]. Due to this, an increased flux of glucose through the polyol pathway will increase the risk of irreversible AGE formation and its associated oxidative stress by raising the levels of fructose and its downstream metabolites, especially glyceraldehyde-3-phosphate (G3P), methylglyoxal (MG), and 3-deoxyglucosone (3-DG) (Figure 5). Such AGE formation can further exacerbate oxidative stress by damaging the enzymes that regenerate antioxidants [135] or by inhibiting the transport molecules that facilitate antioxidant transport [136].

Protein glycation and AGE accumulation are known to be involved in the pathogenesis of diabetic cataracts [137,138]. However, the association between secondary fructose production via the polyol pathway, downstream fructose metabolism, and the effects of resultant oxidative stress on lens protein structure and function in the pathobiology of diabetic cataract has not to date been well studied in the lens. We do know that AGEs derived from the fructose metabolite methylglyoxal (Figure 5), also known as ‘MOLDs’ (methylglyoxal-derived lysine dimers), are increased in pre-cataractous diabetic lenses and are doubled in cataractous lenses [139], demonstrating MOLD accumulation to be in line with cataract progression. In contrast, the metabolic equivalent derived from glucose (glyoxal-derived lysine dimers or ‘GOLDs’) displays a similar trend of association with cataract development [140], but at a level around 10 times lower than that of MOLDs. This is despite glucose levels remaining higher than fructose in diabetic lenses [141], underlining the greater impact of these fructose metabolites. In general, methylglyoxal (MG) is known to increase in diabetes [142]. MG will also lead to oxidative stress by other mechanisms. MG can be detoxified to D-lactate, but this is reliant on GSH [143,144] which is reduced in hyperglycaemia. In the absence of sufficient GSH, MG may be alternately detoxified by Aldo-keto reductases (AKRs), though this requires NADPH as a co-factor which is also reduced in hyperglycaemia [144]. As a result, MG itself will not only further deplete GSH and NADPH levels, but as these become depleted less MG will be detoxified, further exacerbating the irreversible formation of MOLDs. MG also has other detrimental effects in the lens; the incubation of MG with lens alpha-crystallin proteins leads to extensive protein cross linking [145,146], a precursor to protein aggregation and cataract formation.

Another fructose metabolite, glyceraldehyde-3-phosphate (G3P), is also a potent AGE-forming molecule [147,148] and is associated with the development of cataracts in animal models [149]. Since 100% of G3P exists in an open chain state, an equimolar amount of this triose forms 200 times more glycated proteins than glucose [150]. The fructose metabolite 3-deoxyglucosone (3-DG) has also been shown to enhance ROS levels [151]. This is most likely through the inhibition of antioxidant enzymes such as glutathione peroxidase, which normally functions to detoxify H_2_O_2_ and lipid peroxidases [152], and glutathione reductase, preventing the regeneration of GSH from GSSG [153]. Although the mechanism by which 3-DG inhibits these enzymes is not yet known, one potential explanation is the direct modification of these enzymes by glycation. Both fructose and its metabolite MG are shown to directly glycate enzymes [154]. As there is no protein turnover or repair within the anucleate fibre cells, post translational modifications (such as glycation) to antioxidant enzymes would undoubtedly lead to altered function over time. In support of this, it was found that in human lens epithelial cells cultured in high glucose, AGE formation was shown to correlate with the decreased activity of several antioxidant enzymes including superoxide dismutase, catalase, glucose 6-phosphate dehydrogenase, NADP-dependent isocitrate dehydrogenase, and glutathione reductase [135], leading to a reduced antioxidant capacity in the lens.

Taken together, it appears that the increased flux of glucose through the polyol pathway can lead to oxidative stress and protein glycation via a variety of pathways, and that the contributions of these pathways to diabetic cataractogenesis are influenced by species differences in the relative activity of polyol pathway enzymes to favour either sorbitol formation (osmotic stressor) or fructose formation (oxidative stressors). Regardless of the relative contributions from osmotic or oxidative stress, the increased flux of glucose through the polyol pathway ultimately causes a loss of fibre cell volume control that manifests as the diabetic cataract damage phenotype. In the final section, we will discuss how these combined stresses impact on the ability of cortical fibre cells to regulate their volume.

## 7. The Link between Hyperglycaemic Polyol Metabolism and Cell Volume Dysregulation

**Figure 6:**
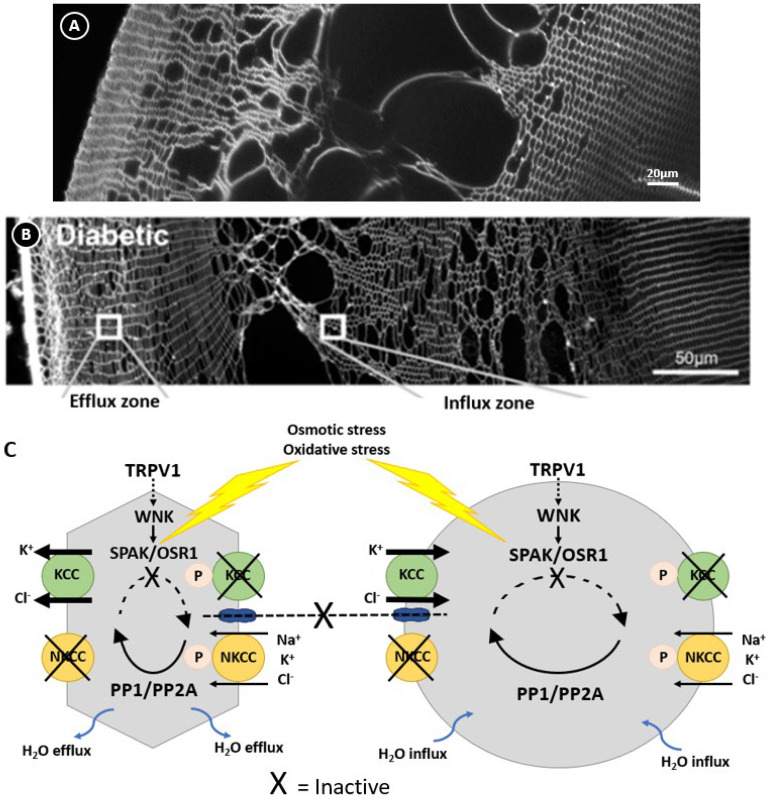
Regulation of lens volume in the normal and diabetic lens. Confocal images comparing the damage phenotypes observed in rat lenses by either organ culturing lenses in NEM (**A**), which oxidises and inactivates SPAK, or obtained from diabetic animals 1 month after streptozotocin injection (**B**). (**C**) Model linking the damage phenotypes observed in NEM-treated (**A**) and diabetic (**B**) lenses where osmotic induced volume changes and oxidative stress inhibit the kinases that regulate KCC and NKCC, causing the peripheral cell shrinkage and deeper fibre cell swelling observed in this zone. Figure (**B**,**C**); Adapted with permission from Donaldson PJ et al. (2017) [45].

The hypothesis that the diabetic cataract damage phenotype is initiated by the dysfunction of volume regulation in fibre cells initially came from rodent studies investigating the activity of cation chloride Acotransporters in fibre cell volume control. Pharmacological modulation of NKCC or KCC activity in organ-cultured rat lenses produced localised changes to differentiating fibre cells in the outer cortex reminiscent of the damage phenotype observed in the STZ model of diabetic cataracts. Rat lenses organ-cultured in isotonic artificial aqueous humour (iso-AAH) with the NKCC-specific inhibitor bumetanide exhibited dilation of the extracellular spaces between fibre cells in the deeper zone of the outer cortex [74], while the KCC-specific inhibitor [(dihydronindenyl)oxy] alkanoic acid (DIOA) resulted in the swelling of fibre cells in the lens periphery with some extracellular space dilations evident in deeper cortical layers [71]. These distinctly different damage phenotypes indicated that, under isotonic conditions, NKCC1 mediates ion influx in the lens cortex, while KCCs mediate ion efflux in peripheral fibre cells and ion influx in the deeper fibre cell layers (Figure 3). While localised to the same region in the lens cortex as the diabetic cataract phenotype, the morphological changes to fibre cells induced by inhibiting either NKCC1 or KCC were not precisely the same as the localised zone of fibre cell swelling seen in the STZ diabetic rat lens. However, a subsequent study used N-Ethylmaleimide (NEM) to activate KCC, again in iso-AAH organ-cultured rat lenses, and this caused a phenotype of cell swelling and tissue liquefaction around 150 μm from the lens surface (Figure 6A), almost identical to that seen in the STZ diabetic rat model (Figure 6B), together with fibre cell shrinkage in the peripheral fibre cell layers [71]. What is of specific interest here is the mechanism of action of NEM, which does not directly activate KCC but in fact inhibits SPAK, the regulatory kinase that would normally ‘switch off’ KCC. NEM is an oxidising reagent and readily oxidises protein thiol groups over a wide pH range [155]. SPAK inactivation by NEM is due to the oxidation of thiol groups within this regulatory kinase [156]. This is direct evidence of the potential for oxidative damage to inhibit the fundamental mechanisms that underpin cell volume regulation and transparency in the lens outer cortex. With this in mind, we have proposed a model (Figure 4) in which both osmotic and oxidative stress resulting from the hyperglycaemic metabolism of glucose in the polyol pathway result in dysfunction of the cell volume regulatory mechanisms, leading to diabetic cataract formation (Figure 6).

## 8. Conclusions

Hyperglycaemia results in the upregulation of the polyol pathway and the generation of sorbitol, fructose, and fructose-derived metabolites, contributing to lenticular oxidative stress and the modification of proteins, including proteins linked to maintaining redox balance and regulating cell volume. The control of water content and movement at both the cellular and organ level is fundamental to the function and transparency of the human lens. For this reason, the lens possesses regulatory mechanisms that respond quickly to osmotic stress to maintain a steady state fibre cell and whole lens volume. However, over time, hyperglycaemia-induced oxidative stress and irreversible protein modifications impair the volume regulation machinery of the lens, resulting in the osmotic cell swelling characteristic of diabetic cortical cataracts.

It is now more widely recognised that it is the synergistic effects of osmotic stress, oxidative stress, and irreversible AGE formation that result in the gradual loss of the homeostatic responses that normally regulate cell volume and redox balance, coinciding with the chronic onset of diabetic cataracts several years after the diagnosis of diabetes. The increased flux of glucose through the polyol pathway results in amplification loops which exacerbate ROS production, increase oxidative stress, increase the formation of AGEs, and ultimately lead to protein damage. However, there are still significant gaps in our knowledge, particularly related to fructose metabolism in the lens and its exact contribution to oxidative stress and osmotic stress. These physiological aspects must be better understood to successfully target therapies for the prevention of diabetic cataracts in humans, but, given that species differences exist in the uptake and metabolism of glucose in the lens, careful consideration is required around the selection of an appropriate animal model. With this in mind, the targeting of a specific glucose and/or fructose metabolic pathway that alleviates oxidative stress and protects the cell volume machinery of the lens may delay the onset of cataracts and avoid the looming cataract epidemic caused by our increasing diabetic population.
